# Full-spectrum cannabidiol-rich oil modulates behavior and neurochemical alterations in a rodent model of maple syrup urine disease

**DOI:** 10.1007/s11011-026-01958-x

**Published:** 2026-09-03

**Authors:** Isabela da Silva Lemos, Sasckia Duarte, Meline Oliveira dos Santos Morais, Carolina Giassi Alano, Laura Schmidt Quinsani, Rafaela Tezza Matiola, Flávia Saccon Niero, Lucas Candido Pedro, Zilli Réus, Flavia Karine Rigo, Rafael Mariano de Bitencourt, Emilio Luiz Streck

**Affiliations:** 1 Laboratório de Doenças Neurometabólicas, Programa de Pós-graduação em Ciências da Saúde, Universidade do Extremo Sul Catarinense, Criciúma, SC 88806-000 Brasil; 2 Universidade do Extremo Sul Catarinense, Criciúma, SC 88806-000 Brasil; 3 Laboratório de Fisiopatologia Experimental, Programa de Pós-graduação em Ciências da Saúde, Universidade do Extremo Sul Catarinense, Criciúma, SC 88806-000 Brasil; 4 Universidade Sul de Santa Catarina, Tubarão, SC 88704-900 Brasil; 5 Faculdade de Medicina, Universidade de Rio Verde, Rio Verde, GO Brasil

**Keywords:** Branched-chain amino acids, Memory, Cholinergic system, Oxidative stress, Cannabinoid, Inborn errors of metabolism

## Abstract

Maple Syrup Urine Disease (MSUD) is caused by a genetic mutation in the branched-chain α-ketoacid dehydrogenase complex, resulting to accumulation of branched-chain amino acids (BCAAs) that affect the central nervous system and cause neurochemical alterations and behavioral changes. In this line, full-spectrum cannabidiol (CBD)-rich oil has emerged as a potential therapeutic strategy. Therefore, this study aims to evaluate the effects of two doses of the compound full-spectrum CBD-rich oil in a BCAA-induced MSUD rat model, against behavioral, cholinergic, inflammatory, and oxidative stress parameters. For this, animals were divided into six groups: control group, CBD 3.5 mg/kg group, CBD 7.5 mg/kg group, BCAA group, BCAA + CBD 3.5 mg/kg group, and BCAA + CBD 7.5 mg/kg group. The treatment was administered over 21 days; after that, the animals were subjected to open-field and object recognition tests. Next, we extracted the cerebral cortex to analyze cholinergic function, inflammation, and oxidative stress. The results show that the open-field test revealed no differences in crossings and rearings across all groups. In object recognition test, control and CBD 3.5 groups showed improved short- and long-term memory compared to training. The CBD 7.5 and BCAA + CBD 3.5 groups showed improvement only in short-term memory. BCAA control and BCAA + 7.5 did not present differences. In the cholinergic system, BCAA control showed decreased choline acetyltransferase (ChAT) activity, which was reversed by CBD treatment at both doses. The BCAA + CBD 7.5 shows increased ChAT activity compared to control group. While acetylcholinesterase (AChE) was reduced in the CBD 7.5 groups and increased in the BCAA control group, both CBDs reversed this increase in BCAA control group. Inflammatory cytokines show increased interleukin-1β in BCAA control group, and the CBD treatment decreases its levels compared to BCAA and saline control groups. Interleukin-6 increases in BCAA control group, and CBD 3.5 reverses it. Tumoral necrosis factor-alpha was reduced in BCAA + CBD 3.5 and BCAA + CBD 7.5 compared to control and BCAA control groups. Further, under oxidative stress, BCAA control increases 2,7-dichlorofluorescein oxidation and thiobarbituric acid levels, which were reversed by CBD treatment. Sulfhydryl content was decreased in CBD 7.5, BCAA control group, BCAA + CBD 3.5, and BCAA + CBD 7.5 compared to control group. Superoxide dismutase activity increased across all groups, whereas catalase activity decreased in the BCAA control group; treatment with CBD 7.5 reversed this reduction. Overall, we conclude that full-spectrum CBD-rich oil shows therapeutic potential for MSUD, although optimal dosing and treatment duration require further investigation.

**Graphical abstract:**

**Figure:**
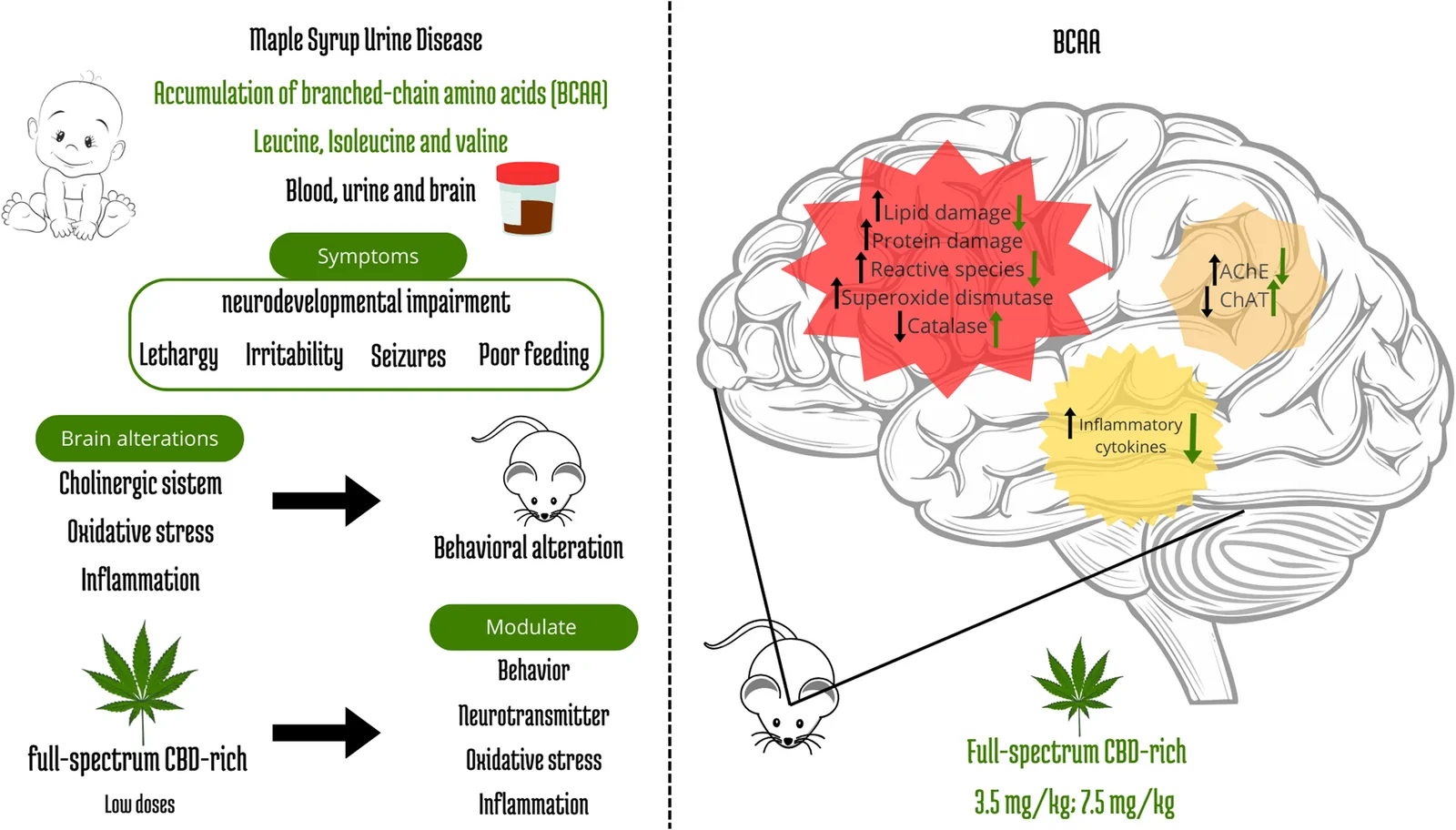

**Supplementary Information:**

The online version contains supplementary material available at https://doi.org/10.1007/s11011-026-01958-x.

## Introduction

Inborn errors of metabolism (IEM) encompass thousands of diseases characterized by altered accumulation, degradation, synthesis, transport, and storage of metabolites, leading to biochemical changes that underlie clinical manifestations. These diseases arise from genetic mutations that affect the central nervous system (CNS). As a result, IEMs present with severe symptomatology (Ziegler et al. 2023). Thus, Maple Syrup Urine Disease (MSUD) involves a failure in the breakdown of branched-chain amino acids (BCAAs) due to mutations in the subunits of the enzymatic complex that metabolize these BCAAs, leading to the accumulation of leucine, isoleucine, and valine (Chuang et al. 2019). Studies show that these BCAAs and their respective metabolites accumulate early in life and, along with a late diagnosis, may result in neurodevelopmental impairment and, in the most severe cases, death within the first week of life (Dancis et al. 1959; Strauss et al. 2020). Furthermore, MSUD may affect 1:185.000 live births. The Mennonite community’s current prevalence is 1:176 live births (Fisher et al. 1991; Puffenberger 2003). Data are estimated in Brazil due to a lack of diagnosis offered by the public health network (Herber et al. 2015).

Regarding this, we know that late diagnostics can lead to irreversible neurological and motor damage, mainly in classical phenotypic patients who have 0–2% branched-chain keto acid dehydrogenase complex activity (Chuang et al. 2019). Vogel and colleagues have shown that dietary restriction, the primary treatment for MSUD, may control BCAA levels in the blood and even reach metabolic control. However, they found changes in brain neurotransmitters, concluding that dietary treatment and metabolic control are insufficient to prevent neurocognitive changes (Vogel et al. 2014). Further, studies exhibit data that the decrease in neutral amino acids in the brain is a result of competition across the blood-brain barrier, which leads to a reduction of neurotransmitter synthesis by lower neutral amino acid levels, implicating neurological dysfunction and the appearance of learning and memory damage (Smith et al. 1987; Wajner et al. 2000).

In this line, our group previously demonstrated that acetylcholine (ACh) levels are altered in MSUD model, a change associated with neurological damage and behavioral changes in this model. ACh acts in the CNS and is involved in motor control, cognition, and learning, acting across many brain areas, including the cerebral cortex, hippocampus, and forebrain (Huang et al. 2022; Sam and Bordoni 2025). Also, studies present a relationship between the decrease in ACh and cognition and memory problems observed in neurodegenerative diseases (Gold 2003; Haam and Yakel 2017) and in animal models of MSUD, like rats and zebrafish (Lemos et al. 2023, 2024; Scaini et al. 2012a; Wessler et al. 2019). In addition, the exposure to leucine leads to cholinergic dysfunction and is related to anxiety-like behavior presented by zebrafish (Wessler et al. 2019). Although treatment-mediated metabolic control is maintained, it cannot prevent neurocognitive damage; therefore, new approaches are necessary to address neurocognitive impairments in MSUD.

Herein, the BCAA-administration model used, which is not a genetic model, reflects the clinical. Findings in MSUD patients, particularly regarding the neurochemical, inflammatory, and oxidative alterations associated with the disease. Clinical studies have demonstrated that patients with MSUD present increased oxidative damage, including elevated lipid peroxidation and impaired antioxidant defenses, supporting oxidative stress as an important component of disease pathophysiology (Barschak et al. 2008a, b; Mescka et al. 2015b). In addition, inflammatory alterations have been described in patients, with increased circulating levels of pro-inflammatory cytokines such as interleukin-1β (IL-1β) and interlukin-6 (IL-6), which correlate with markers of oxidative damage (Mescka et al. 2015a; Scaini et al. 2018). Similarly, experimental studies in neonates with BCAA administration have reproduced these findings, showing increased reactive species production, lipid peroxidation, and neuroinflammation in brain tissue (Lemos et al. 2024; Sitta et al. 2014; Wessler et al. 2020). Therefore, although the BCAA model is chemically induced rather than genetic, it reproduces key biochemical and inflammatory features observed in the human disease, supporting its translational relevance for investigating mechanisms of damage and evaluating potential therapeutic strategies.

Cannabis-based products have emerged as a new class of drugs with potential therapeutic effects across a broad range of neurodegenerative and psychiatric disorders. Although the mechanisms underlying cannabinoid-induced neuroprotection are not fully elucidated, several studies demonstrate their interaction with multiple targets, including elevation of brain-derived neurotrophic factor (BDNF) levels (Lorenzetti et al. 2023; Pagano et al. 2022), reduction of microglial activation, attenuation of pro-inflammatory mediators, decrease in neuronal death, and facilitation of hippocampal neurogenesis (Atalay et al. 2019; Hickey et al. 2024).

Importantly, recent evidence has shown that full-spectrum *Cannabis sativa* extracts exhibit superior therapeutic effects compared to isolated cannabidiol (CBD). In a model of low-grade inflammation, repeated administration of a full-spectrum CBD-rich preparation reversed depressive-like behavior and hypolocomotion more effectively than purified CBD, an effect attributed to the synergistic interaction among cannabinoids, terpenes, and flavonoids, commonly referred to as the “entourage effect”, which enhances the modulation of inflammatory and oxidative pathways (Ribeiro de Novais Júnior et al. 2024). This growing body of evidence supports the notion that full-spectrum formulations may provide broader neurobiological benefits at lower doses. Moreover, Cannabis-based products are known to modulate neurotransmitter systems, including ACh. Central cholinergic neurotransmission plays a fundamental role in cognitive functions, including learning and memory formation (Lorenzetti et al. 2023). However, the specific effects of orally administered cannabis-based formulations on short-term memory (STM) and long-term memory (LTM), and the involvement of the cholinergic system in these processes, remain unclear. Considering that both memory performance and cholinergic function are impaired in MSUD, understanding how cannabinoids influence these systems is particularly relevant. To this end, the present study aimed to evaluate the effects of two different doses of the compound full-spectrum CBD-rich oil on memory, cholinergic, inflammatory cytokines, and oxidative stress parameters in a rat model of MSUD.

## Materials and methods

### Animals

One hundred and eight male Wistar rats at seven days old were obtained from the Central Animal House of the Universidade do Extremo Sul Catarinense (UNESC). Only male animals were included in the present study to minimize variability associated with hormonal fluctuations during the estrous cycle, which may influence neurochemical, behavioral, and inflammatory responses. These animals were maintained with their mother until day 21, caged, fed ad libitum, and provided with water, with a 12 h light/dark cycle in a controlled-temperature room at 23 ± 1 °C. All procedures were performed in accordance with the Principles of the National Institutes of Health Guide for the Care and Use of Laboratory Animals. The Ethics Committee previously approved this work under protocol number 67/2021.

### BCAA protocol and CBD treatment

After birth, animals were randomized within each cage into a control group, a CBD 3.5 group, a CBD 7.5 group, a BCAA group, a BCAA + CBD 3.5 group, and a BCAA + CBD 7.5 group. This was performed to reduce cage biases across experimental groups. The control group received saline solution (0.9%) subcutaneously and Medium-Chain Triglyceride oil (MCT^®^, Vitafor) via gavage. The full-spectrum CBD-rich oil (HealthyCann, USA) used in this study is a hemp-derived extract containing CBD as the primary cannabinoid together with minor cannabinoids, terpenes, flavonoids, and other phytochemicals naturally present in *Cannabis sativa*. The formulation contains 1.500 mg of CBD, standardized to 50 mg/mL, dissolved in medium-chain triglyceride (MCT) oil. According to the Certificate of Analysis provided by the manufacturer, the oil contains 5.451% CBD (1.526 mg per container) and 0.177% Δ9-Tetrahidrocanabinol (THC), together with minor cannabinoids including CBG (0.072%), CBN (0.024%), CBDV (0.048%), and CBC (0.260%). Terpene composition was evaluated using gas chromatography–mass spectrometry (GC–MS). According to the Certificate of Analysis, the product also passed testing for heavy metals, residual solvents, pesticides, microbials, mycotoxins, and other contaminants within accepted safety limits. The complete Certificate of Analysis is provided as Supplementary Material to ensure reproducibility of the phytochemical composition used in the present study. Because the phytochemical extract was dissolved in medium-chain triglyceride (MCT) oil derived from coconut oil, the same MCT vehicle was administered to the control groups, ensuring that the lipid carrier background was experimentally controlled across treatments.

The doses of CBD used in this study (3.5 and 7.5 mg/kg) were selected based on previous preclinical studies investigating the neuroprotective, anti-inflammatory, and behavioral effects of CBD in rodent models, including studies from our research group demonstrating beneficial effects of CBD in experimental models of neuroinflammation and neuropsychiatric disorders (de Souza Stork et al. 2025; Mori et al. 2017, 2021; Ribeiro de Novais Júnior et al. 2024). These doses fall within the range commonly used in experimental studies evaluating the effects of CBD on neuroinflammation, oxidative stress, and behavioral outcomes. In addition, the use of two doses was intended to explore possible dose-dependent effects of the full-spectrum CBD-rich oil in MSUD model. Using the standard allometric scaling method based on body surface area (FDA USFADA 2005), the doses used in the present study correspond to approximately 0.57 mg/kg and 1.22 mg/kg in humans, which would represent approximately 40 mg and 85 mg, respectively, for a 70-kg adult. These values fall within the range of oral CBD doses previously investigated in clinical studies and reported to be safe and well tolerated (20–600 mg/day) (Iffland and Grotenhermen 2017). Therefore, the doses tested in this study are within a realistic pharmacological range and may be considered relevant for potential translational interpretation.

**Fig. 1:**
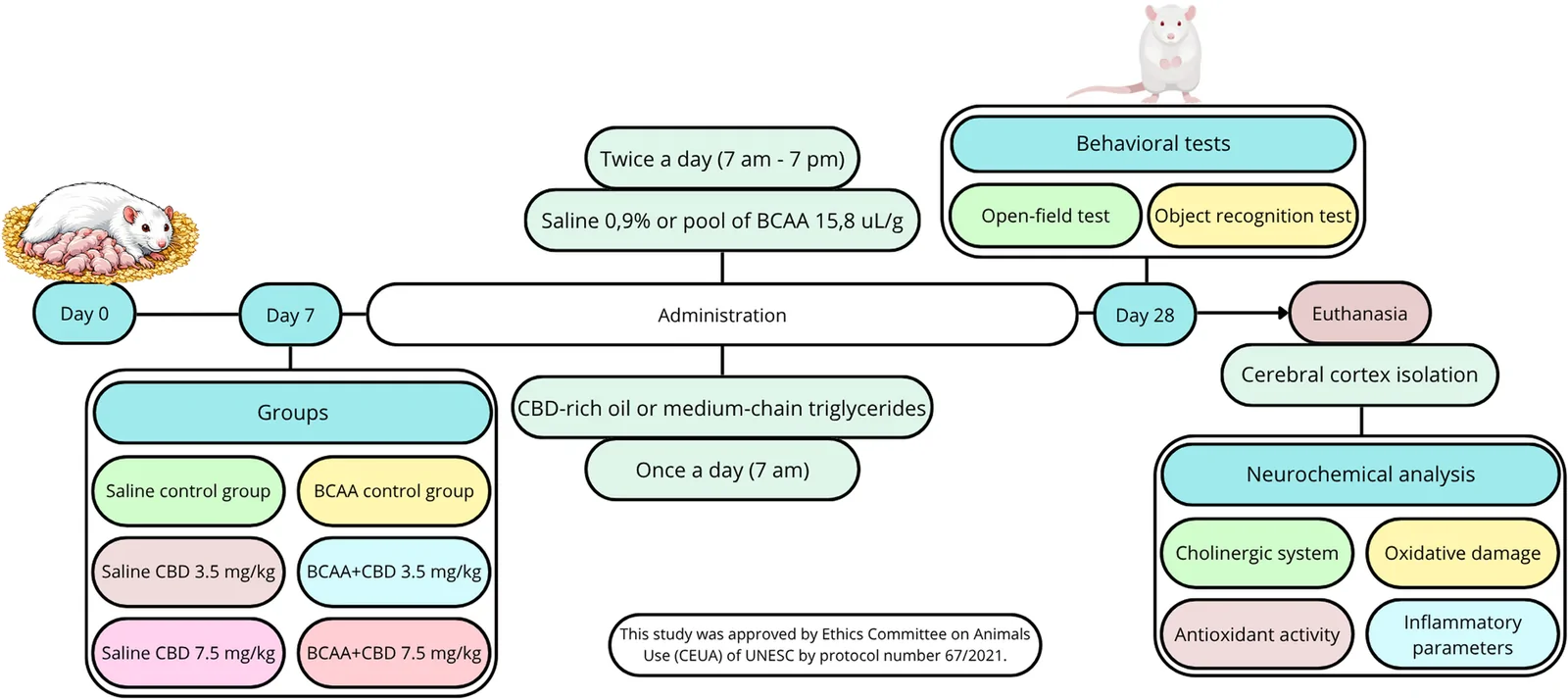
Experimental design

The CBD 3,5 received saline solution (0.9%) subcutaneously, and CBD 3.5 mg/kg via gavage. CBD 7.5 received saline solution (0.9%, MED FLEX^®^, Eurofarma - SP) subcutaneously and CBD 7.5 mg/kg via gavage. The BCAA group received a pool (15.8 uL/g) of leucine (190 mmol/L, L8000, Sigma-Aldrich), isoleucine (59 mmol/L, I2752, Sigma-Aldrich), and valine (69 mmol/L, V0500, Sigma-Aldrich) subcutaneously, and MCT oil via gavage. BCAA + CBD 3.5 received a pool of BCAA (15.8uL/g) subcutaneously and CBD 3.5 mg/kg via gavage. BCAA + CBD 7.5 received a pool of BCAA (15.8uL/g) subcutaneously and CBD 7.5 mg/kg via gavage. Subcutaneous administration was twice daily at a volume of 15 µL/g body weight, and this administration volume was standardized across all experimental groups. Gavage administration was once daily, beginning at 0.1 mL per animal at a body weight of 18 g at the start of the experiment, and continued until around 150 g, with a volume standardized across all groups. BCAA pool was dissolved in saline (0.9%) (Bridi et al. 2006; Scaini et al. 2012b). CBD doses were diluted in MCT oil. After 21 days of administration, behavioral tests were performed, and the entire cerebral cortex was isolated to evaluate the cholinergic system, oxidative stress parameters, and inflammatory cytokines, as shown in Fig. 1. A total of 108 animals were used, divided into 6 groups of *n* = 18. The number is justified once the 10–18 animals per group have completed the behavioral tests; the same animals were used for these analyses, given that the test does not affect biochemistry. For this, *n* = 6 was used for the cholinergic system, *n* = 6 for the inflammatory parameters, and *n* = 6 for the oxidative stress analysis, given that the homogenization procedures differ across techniques, for a total of *n* = 18 per group.

### Behavioral test

Following treatment, animals from all groups underwent behavioral testing. The open field test was performed first to assess spontaneous locomotor activity and general exploratory behavior in a low-stress, noninvasive context. This step is particularly important to rule out potential motor impairments that could confound the interpretation of performance in subsequent cognitive tasks. The object recognition memory test was conducted afterward, as it relies on the animal’s natural exploratory behavior and is minimally stressful.

### Open field test

This test was used to evaluate the spontaneous locomotor activity in both animal models receiving BCAA and CBD. The open field test was performed 12 h after the last administration. Rats (*n* = 10–18 per group) were placed in the open-field apparatus to assess spontaneous locomotor activity. The apparatus consisted of an arena measuring 40 × 60 cm, surrounded by 50 cm-high brown plywood walls and a glass front wall, for spontaneous activity. The floor of the open field was divided into nine rectangles (15 × 20 cm each) by black lines (Réus et al. 2017). Animals were gently placed in the left rear quadrant and allowed to explore the arena for 5 min. An expert observer counted the number of horizontal (crossing) and vertical (rearing) activities performed by each rat during the 5-minute observation period.

### Object recognition test

The object recognition test was taken using the same apparatus as the open-field test. The object recognition test is a widely used behavioral task in neuroscience and psychology to evaluate recognition memory, a type of declarative memory. The same animals (*n* = 10–18 per group) underwent a habituation session in which they freely explored the open field for 5 min without objects. Twenty-four hours after habituation, training was conducted by placing the individual rats in the apparatus for 5 min, during which two identical objects (A1 and A2, both cubes) were positioned in two adjacent corners, 10 cm from the walls. 1.5 h after the training was performed, the STM recognition memory test was performed; in STM, one familiar object (A2) was replaced by a new object (B, a rectangle), and the rats explored the open field for 5 min. Also, 24 h after the STM, LTM recognition memory test was conducted, in which object B was replaced with a novel object (C, a pyramid with a square base). The rats were then placed in the open field for 5 min. All objects shared similar textures (smooth), colors (blue), and sizes (150–200 g), but differed in shape. During the training phase, object preference was assessed to detect potential baseline biases. The preference index in the training section was calculated as (time exploring one object / total exploration time) × 100. A recognition index was calculated for each animal during the test session. It reports the ratio TB/(TA + TB) (TA = time spent exploring the familiar object A; TB = time spent exploring the novel object B), and it reports the ratio TC/(TA + TC) (TA = time spent exploring the familiar object A; TC = time spent exploring the novel object C). Between trials, the objects were washed with a 10% ethanol solution. Exploration was defined as sniffing (exploring the object 3–5 cm away from it) or touching the object with the nose or forepaw (Vianna et al. 2001). The criterion adopted in the present study was selected to capture subtle exploratory behaviors while maintaining specificity for active investigation and was applied consistently across all experimental groups.

### Acetylcholinesterase activity

Samples were homogenized 1:10 in 10 mM potassium phosphate buffer (P5379; P3786, Sigma-Aldrich), pH 7.5, and centrifuged at 10,000 rcf for 10 min at 4 °C to remove cell debris. The supernatants were separated to determine protein content and Acetylcholinesterase (AChE) activity.

AChE activity was measured by the protocol of Ellman and colleagues using 150 mM potassium phosphate buffer (P5379; P3786, Sigma-Aldrich) and 10 mM 5,5′-dithiobis (2-nitrobenzoic acid) (DTNB, D8130, Sigma-Aldrich), incubated with 10 µg of the sample (previously homogenized as mentioned above, pH 7.5) for 3 min at 25 °C. In this mixture, 8 mM acetylthiocholine (A5751, Sigma-Aldrich) was added, and the mixture was read at 420 nm in a *SpectraMax M3* at 37 °C for 5 min. The results were shown as µmol ACSCh/h/mg of protein (Ellman et al. 1961).

### Choline acetyltransferase activity

Samples were homogenized 1:20 in 0.5 M sodium phosphate buffer (10049-21-5; 7558-79–4, Exodo Científica), pH 7.2, and centrifuged at 3000 rpm for 10 min at 4 °C to remove cell debris. The supernatants were separated to determine protein content and Choline acetyltransferase (ChAT).

ChAT activity was performed according to the procedure of Chao and colleagues using 0.5 mM sodium phosphate buffer, 3 M sodium chloride (1528-1, Dinâmica Química Contemporânea Ltda), 1.1 mM EDTA (E6758, Sigma-Aldrich), 0,76 mM neostigmine sulfate (N2126, Sigma-Aldrich), 25 µg sample, 1 mM 4,4’-ditiopiridine (143049, Sigma-Aldrich), 1 M choline chloride (CC07538RA – Exodo cientifica), and water, incubated for 5 min at 37 °C. After that, 6.2 mM acetyl-coenzyme A (A2181, Sigma-Aldrich) was added, and the mixture was read in a SpectraM*ax M3* for 20 min at 324 nm. The results were expressed as nmol/min/ug of protein using the coefficient 1.98 × 10^4 (Chao and Wolfgram 1973).

### Sample preparation for the determination of inflammatory and oxidative stress parameters

After the behavioral test, animals were killed by decapitation, and the brain was excised on a Petri dish placed on ice. The cerebral cortex was isolated and stored at -80 °C for analysis. The tissue was weighed and homogenized in 1:10 (w/v) of ice-cold 20 mM sodium phosphate buffer (10049-21-5; 7558-79–4, Exodo Científica) + 140 mM potassium chloride (P9541, Sigma-Aldrich), pH 7.4. Homogenates were centrifuged at 3.500 rpm for 10 min at 4 °C to remove cellular debris. The supernatants were separated to determine oxidative stress and inflammatory parameters. Protein was analyzed using serum albumin as the standard, according to the method of Lowy et al. (1951) described below (Lowry et al. 1951).

### Inflammation

The levels of the pro-inflammatory cytokines IL-1β, IL-6, and TNF-α were evaluated by an enzyme-linked immunoabsorbent assay (DuoSet ELISA) of capture (R&D Systems), according to manufacturer instructions. Absorbance was measured at 450 nm. Data were expressed as pg/mg of protein.

### Thiobarbituric acid reactive substances (TBARS)

Lipid damage was evaluated by TBARS levels, as described by Esterbauer and Cheeseman (1990). A calibration curve was prepared using 1,1,3,3-tetramethoxypropane (108383, Sigma-Aldrich). 100 µl were treated with 0.67% TBA (T5500, Sigma-Aldrich) and 10% TCA (1072-1, Dinâmica Química Contemporânea Ltda). This mixture was boiled for 2 h, and after cooling, butanol (P1000510060081, Dinâmica Química Contemporânea Ltda) was added. The samples were mixed and centrifuged at 5000 rpm for 3 min. The upper phase containing the colored product was measured fluorometrically at 515 nm (excitation) and 553 nm (emission). Results were expressed as nmol of TBARS/mg of protein (Esterbauer and Cheeseman 1990).

### 2’,7’-Dichlorofluorescein (DCFH) oxidation

Reactive oxygen and nitrogen species production was analyzed by the method of 2’,7’-DCFH oxidation (LeBel et al. 1992). Samples (30 µL) were incubated with homogenization buffer (30 µL) and 1.0 mM 2’,7’-dichlorofluorescein diacetate (DCFH-DA, 287810, Sigma-Aldrich) solution (240 µL) in the dark for 30 min at 37 °C. The fluorescence was measured at 488 nm (excitation) and 525 nm (emission). Results were shown as nmol DCF/mg of protein (LeBel et al. 1992).

### Sulfhydryl content

Protein damage was evaluated by measuring sulfhydryl content using the assay of Aksenov and Markesbery (2001). Samples (22 µL), 0.1 M phosphate buffer, and the reagent 10mM 5,5’-dithio-bis (2-nitrobenzoic acid) (DTNB, D8130, Sigma-Aldrich) were incubated for 15 min. Absorbance was read at 412 nm. Results were expressed as nmol TNB/mg of protein (Aksenov and Markesbery 2001).

### Antioxidant enzyme activities

The activities of superoxide dismutase (SOD) and catalase (CAT) were assessed by the methods of Bannister and Calabrese (1987) and Aebi (1984), respectively. SOD activity was assayed using 50 µL of sample, 50 mM glycine buffer (H1610-0718, BioRad) (pH 10.2), 0.1 mM CAT (C9322, Sigma-Aldrich), and 1 mM epinephrine (E4250, Sigma-Aldrich) at 32 °C for 20 min at 480 nm. CAT activity was performed in a reaction medium containing samples (20 µL), 20 mM hydrogen peroxide Dinâmica Química Contemporânea Ltda), 0.1% Triton X-100 (T9284, Sigma Aldrich), and 10 mM potassium phosphate buffer (P5379; P3786, Sigma-Aldrich), pH 7.0. Absorbance was read at 240 nm. The results of both activities were expressed as units/mg of protein (Aebi 1984; Bannister and Calabrese 1987).

### Protein assay

After homogenization, the supernatants from the samples were used for protein assay using the Lowry method. For this purpose, bovine serum albumin is used as a standard, and the process is based on Folin’s phenol reagent, which reacts with proteins in the sample in the presence of Cu^2+^ ions. For the standard curve, 1 mg/mL bovine albumin (A9647, Sigma-Aldrich) was used with 1000 µL reactive C (reactive A (1702-1, Dinâmica Química Contemporânea Ltda; C2011.01.AH, Labsynth), B1 (1129, Dinâmica Química Contemporânea Ltda), and B2 (P.10.0972.009.03.30, Dinâmica Química Contemporânea Ltda) at 100:1:1 and water, for a final volume of 1.300 µL. 10 µL of the sample, 1000 µL reactive C, and 190 µL of water were also used, and this mixture was incubated for 10 min. After that, 100 µL of Folin’s phenol 1 N (2326, Dinâmica Química Contemporânea Ltda) was added to all the samples, the standard curves, and the mixture, and the mixture was incubated in the dark for 20 min. We measured absorbance at 650 nm on a SpectraM*ax M3* at room temperature (Lowry et al. 1951). Data were expressed as mg of protein.

### Statistical analysis

The statistical analysis was performed using two methods, depending on the results. The Shapiro–Wilk normality test was applied to evaluate the normality of the distribution. Behavioral parameters were presented non-parametrically and analyzed using the Wilcoxon test. For the parametric tests, a one-way analysis of variance (ANOVA) was used, and the data were expressed as mean ± Standard Error of the Mean (SEM). The open field test was analyzed using one-way ANOVA followed by Tukey’s post hoc test. The object recognition test was analyzed using a paired-samples t-test to evaluate differences between the training and test sessions. Data were expressed as mean ± SEM. We used the Statistical Package for the Social Sciences (SPSS, 31.0). Otherwise, neurochemical data that met the assumptions of normality and homogeneity of variance were analyzed as parametric data and are presented as mean ± standard deviation (SD). Neurochemical analyses of the cholinergic system, oxidative stress, and inflammatory markers were performed using a two-way ANOVA, with experimental condition (Saline or BCAA) and treatment (the respective pharmacological treatment groups) as the two independent factors, followed by Tukey’s post hoc test for multiple comparisons. All results were considered statistically significant at *p* < 0.05. GraphPad Prism software (version 10.3.1) was used to perform the statistical analyses and generate the graphs.

## Results

### Effect of CBD-enriched oil on behavioral tests in the BCAA model

**Fig. 2:**
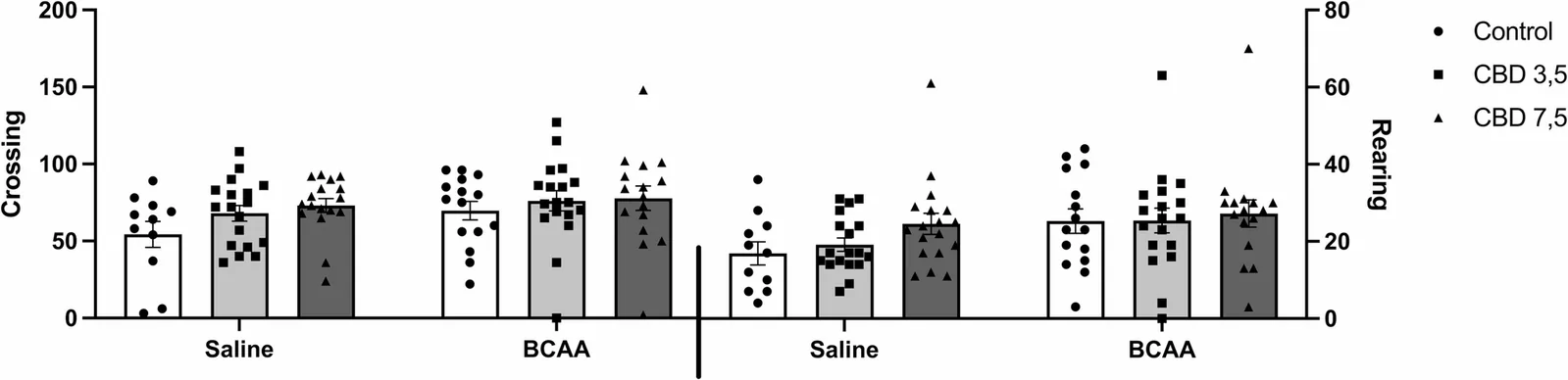
Effects of BCAA administration and CBD treatment on open-field test in rats, showing the number of crossings and rearings (*n* = 10–18). Data are expressed as mean ± Standard Error of the Mean (SEM). *p < 0.05 vs. training session

The first behavioral test performed was the open-field test to evaluate locomotor and exploratory behavior. As shown in Fig. 2, no significant differences were observed among the groups in the number of crossings (*p* = 0.290) or rearings (*p* = 0.212), indicating that the treatments did not alter spontaneous locomotor or exploratory activity.

**Fig. 3:**
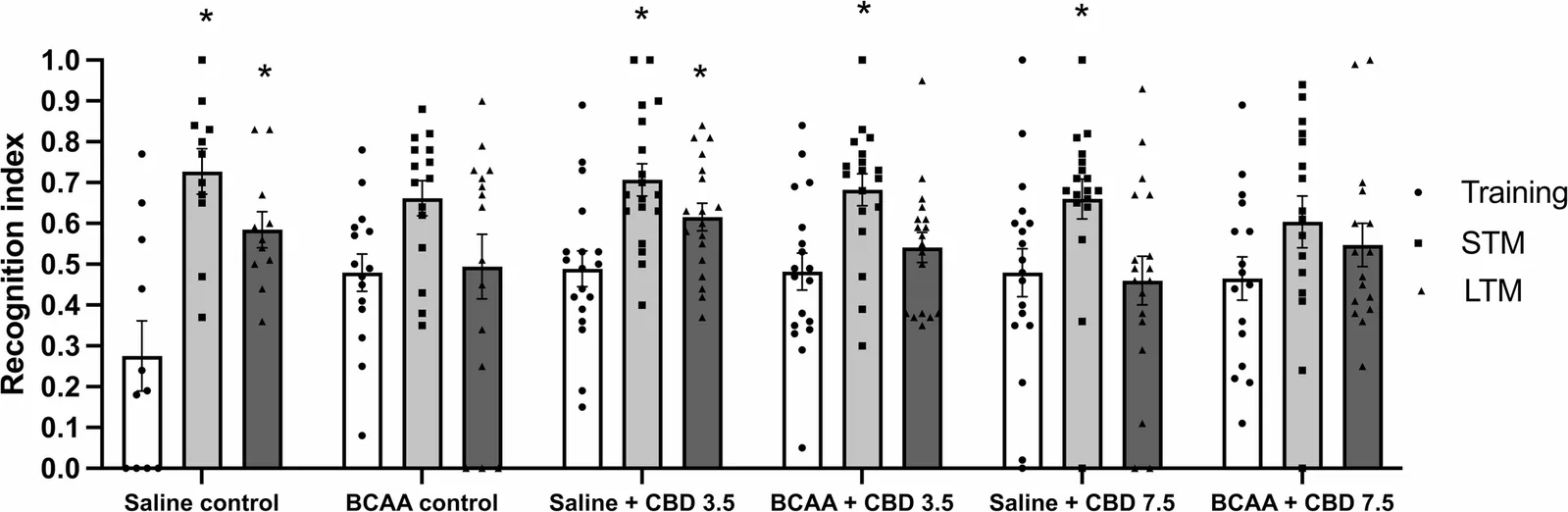
Effects of BCAA administration and CBD treatment on memory by recognition object test in rats, showing the training session, short-term memory (STM), and long-term memory (LTM) (*n* = 10–18). Data are expressed as mean ± Standard Error of the Mean (SEM). *p < 0.05 vs. training session

The second behavioral test evaluated recognition indices associated with short-term memory (STM; 1.5 h after training) and long-term memory (LTM; 24 h after training). During the training session, no differences were observed among the groups (*p* = 0.172), confirming comparable baseline performance. As shown in Fig. 3, the Control and CBD 3.5 groups exhibited significant improvements in both STM (*p* = 0.001 and *p* = 0.003, respectively) and LTM (*p* = 0.005 and *p* = 0.033, respectively) compared with their respective training sessions. Significant improvements in STM were also observed in the CBD 7.5 (*p* = 0.040) and BCAA + CBD 3.5 (*p* = 0.011) groups. In contrast, the BCAA group showed no significant differences in either STM (*p* = 0.570) or LTM (*p* = 0.223), and the BCAA + CBD 7.5 group also did not exhibit significant changes in STM (*p* = 0.190) or LTM (*p* = 0.271) compared with their respective training sessions.

### Effect of CBD-enriched oil on the cholinergic system in the BCAA model

**Fig. 4:**
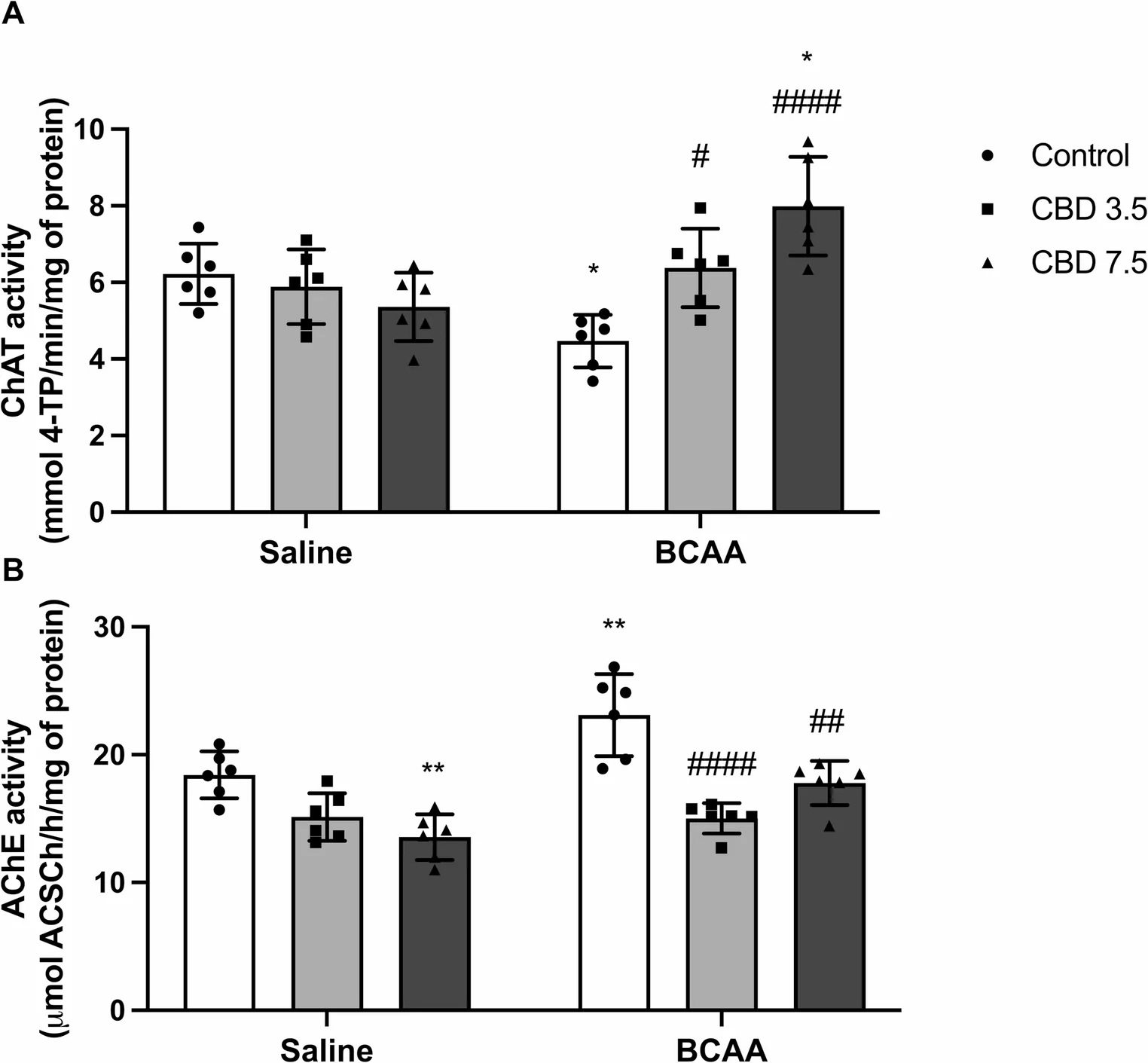
Effects of BCAA administration and CBD treatment on the cholinergic system in rat cortex. (**A**) Choline acetyltransferase (ChAT) activity. (**B**) Acetylcholinesterase (AChE) activity. Experimental groups included saline control, saline CBD 3.5 mg/kg, saline CBD 7.5 mg/kg, BCAA control, BCAA + CBD 3.5 mg/kg, and BCAA + CBD 7.5 mg/kg. Data are presented as mean ± standard deviation (SD), with *n* = 6 animals per group. Statistical analysis was performed using ordinary two-way ANOVA followed by Tukey’s multiple-comparisons test, *p* < 0.05, *p < 0.01, **p < 0.001, ***p < 0.0001 vs. saline control group; #*p* < 0.05, ##*p* < 0.01, ###*p* < 0.001, ####*p* < 0.0001 vs. BCAA control group

After the behavioral evaluation, we assessed the cholinergic system because of its well-established role in memory consolidation. As shown in Fig. 4A, ChAT activity was significantly affected by the interaction between experimental condition and treatment (*p* < 0.0001), whereas no significant effect of the experimental condition alone was observed (*p* = 0.1653). A significant treatment effect was detected (*p* = 0.0073). The BCAA group showed reduced ChAT activity compared with the control group (*p* = 0.0479). Treatment with both CBD doses restored ChAT activity compared with the BCAA group (*p* = 0.0192 and *p* < 0.0001, respectively). Moreover, the BCAA + CBD 7.5 group exhibited higher ChAT activity than the control group (*p* = 0.0360). Figure 4B shows the results for AChE activity, the enzyme responsible for acetylcholine degradation. A significant interaction between experimental condition and treatment (*p* = 0.0125), as well as significant effects of experimental condition (*p* = 0.0001) and treatment (*p* < 0.0001), were observed. Compared with the control group, the CBD 7.5 group exhibited reduced AChE activity (*p* = 0.0019), whereas the BCAA group showed increased AChE activity (*p* = 0.0047). Both CBD doses restored AChE activity in the BCAA-treated animals (*p* < 0.0001 and *p* = 0.0024, respectively).

### Effect of CBD-enriched oil on inflammation in the BCAA model

**Fig. 5:**
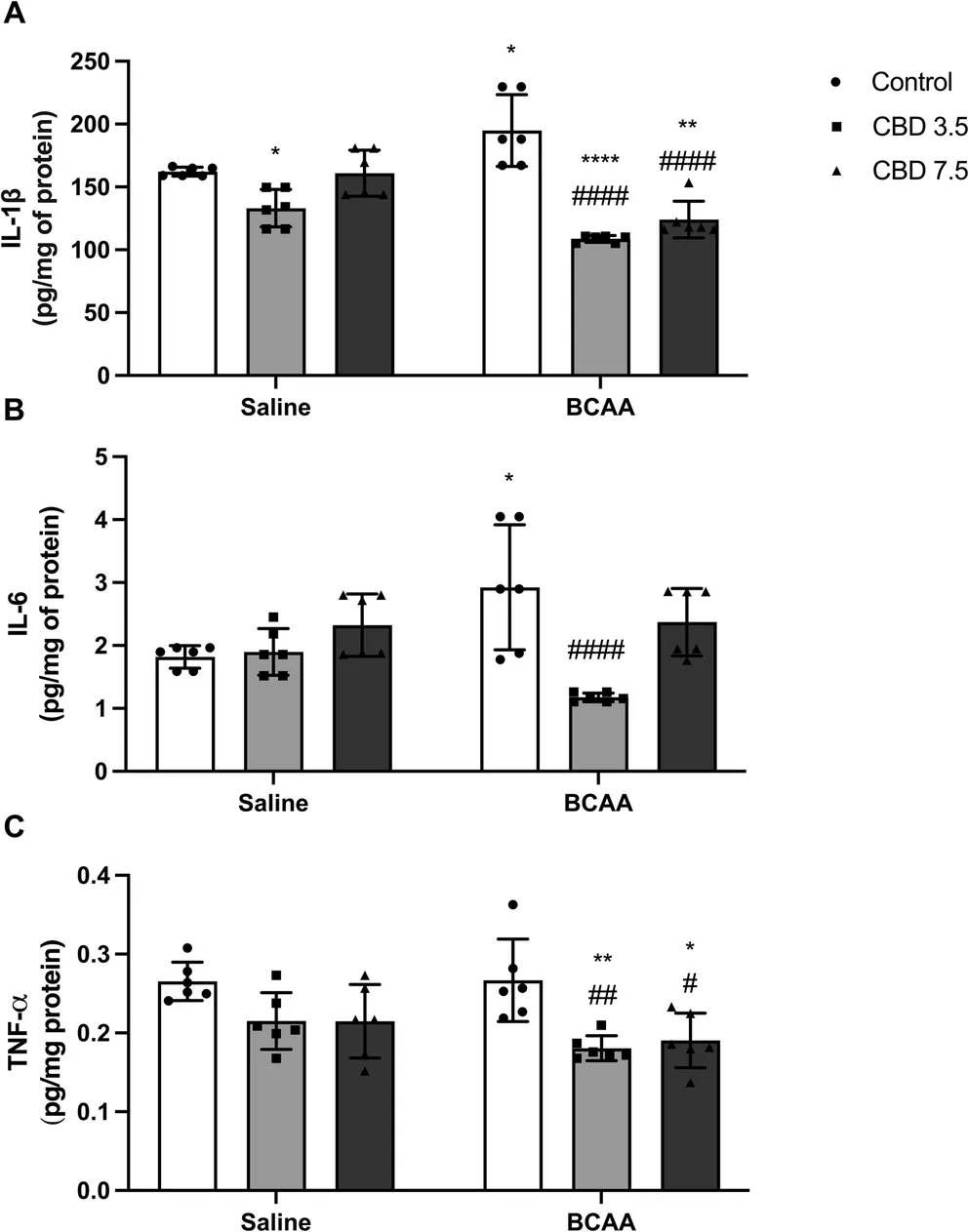
Effects of BCAA administration and CBD treatment on inflammatory parameters in rat cortex. (**A**) Interleukin-1β (IL-1β) levels. (**B**) Interleukin-6 (IL-6) levels. (**C**) Tumor necrosis factor-alpha (TNF-α) levels. Experimental groups included saline control, saline CBD 3.5 mg/kg, saline CBD 7.5 mg/kg, BCAA control, BCAA + CBD 3.5 mg/kg, and BCAA + CBD 7.5 mg/kg. Data are presented as mean ± standard deviation (SD), with *n* = 6 animals per group. Statistical analysis was performed using ordinary two-way ANOVA followed by Tukey’s multiple-comparisons test,, *p* < 0.05, *p < 0.01, **p < 0.001, ***p < 0.0001 vs. saline control group; #*p* < 0.05, ##*p* < 0.01, ###*p* < 0.001, ####*p* < 0.0001 vs. BCAA control group

In this line, we analyzed inflammatory cytokines. As shown in Fig. 5A, IL-1β exhibited a significant interaction effect (*p* < 0.0001), no significant experimental condition effect (*p* = 0.0903), and a significant treatment effect (*p* < 0.0001). Higher IL-1β levels were observed in the BCAA control group than in the control group (*p* = 0.0179). The CBD 3.5 group also showed lower IL-1β levels than the control group (*p* = 0.0455). In the BCAA-treated animals, both CBD doses reduced IL-1β levels compared with the saline-treated groups (*p* < 0.0001 and *p* = 0.0042, respectively) and compared with the BCAA control group (*p* < 0.0001 for both comparisons).

Figure 5B shows that IL-6 exhibited a significant interaction effect (*p* = 0.0009), no significant experimental condition effect (*p* = 0.4206), and a significant treatment effect (*p* = 0.0006). Higher IL-6 levels were observed in the BCAA group than in the control group (*p* = 0.0129). Treatment with CBD 3.5 reversed this increase in the BCAA group (*p* < 0.0001). Figure 5C shows the results for TNF-α levels, which exhibited no significant interaction effect (*p* = 0.4844) or experimental condition effect (*p* = 0.1318), but a significant treatment effect (*p* = 0.0001). TNF-α levels were lower in the BCAA + CBD 3.5 (*p* = 0.0052) and BCAA + CBD 7.5 (*p* = 0.0168) groups than in the control group. Likewise, both treatment groups showed lower TNF-α levels than the BCAA control group (*p* = 0.0044 and *p* = 0.0144, respectively).

### Effect of CBD-enriched oil on oxidative stress in the BCAA model

**Fig. 6:**
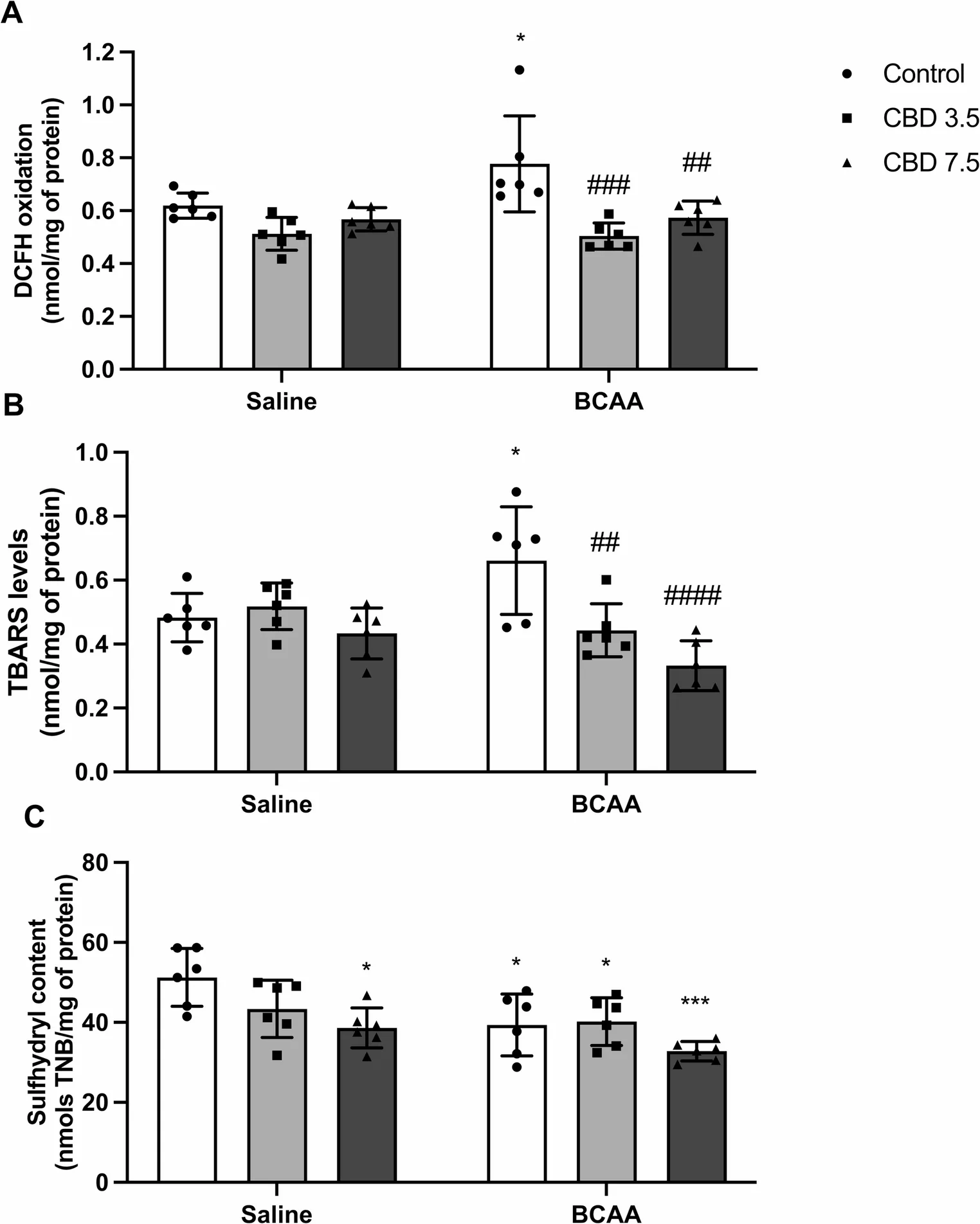
Effects of BCAA administration and CBD treatment on oxidative damage in rat cortex. (**A**) DCFH oxidation. (**B**) TBARS levels. (**C**) Sulfhydryl content. Experimental groups included saline control, saline CBD 3.5 mg/kg, saline CBD 7.5 mg/kg, BCAA control, BCAA + CBD 3.5 mg/kg, and BCAA + CBD 7.5 mg/kg. Data are presented as mean ± standard deviation (SD), with *n* = 6 animals per group. Statistical analysis was performed using ordinary two-way ANOVA followed by Tukey’s multiple-comparisons test,, *p* < 0.05, *p < 0.01, **p < 0.001, ***p < 0.0001 vs. saline control group; #*p* < 0.05, ##*p* < 0.01, ###*p* < 0.001, ####*p* < 0.0001 vs. BCAA control group

Regarding oxidative stress, we evaluated DCFH oxidation, TBARS levels, sulfhydryl content, SOD activity, and CAT activity. As shown in Fig. 6A, DCFH oxidation exhibited no significant interaction effect (*p* = 0.0548) or experimental condition effect (*p* = 0.0902), but a significant treatment effect (*p* < 0.0001). DCFH oxidation was increased in the BCAA control group compared with the control group (*p* = 0.0467), and both CBD treatment doses reversed this increase (*p* = 0.0001 and *p* = 0.0052, respectively).

Figure 6B shows that TBARS levels exhibited a significant interaction effect (*p* = 0.0026), no significant experimental condition effect (*p* = 0.9815), and a significant treatment effect (*p* = 0.0003). TBARS levels were increased in the BCAA control group compared with the control group (*p* = 0.0418), whereas both CBD doses reversed this increase (*p* = 0.0075 and *p* < 0.0001, respectively). As shown in Fig. 6C, sulfhydryl content exhibited no significant interaction effect (*p* = 0.2258), but significant effects of experimental condition (*p* = 0.0020) and treatment (*p* = 0.0024). Sulfhydryl content was reduced in the CBD 7.5 (*p* = 0.0153), BCAA control (*p* = 0.0255), BCAA + CBD 3.5 (*p* = 0.0443), and BCAA + CBD 7.5 (*p* = 0.0002) groups compared with the control group.

**Fig. 7:**
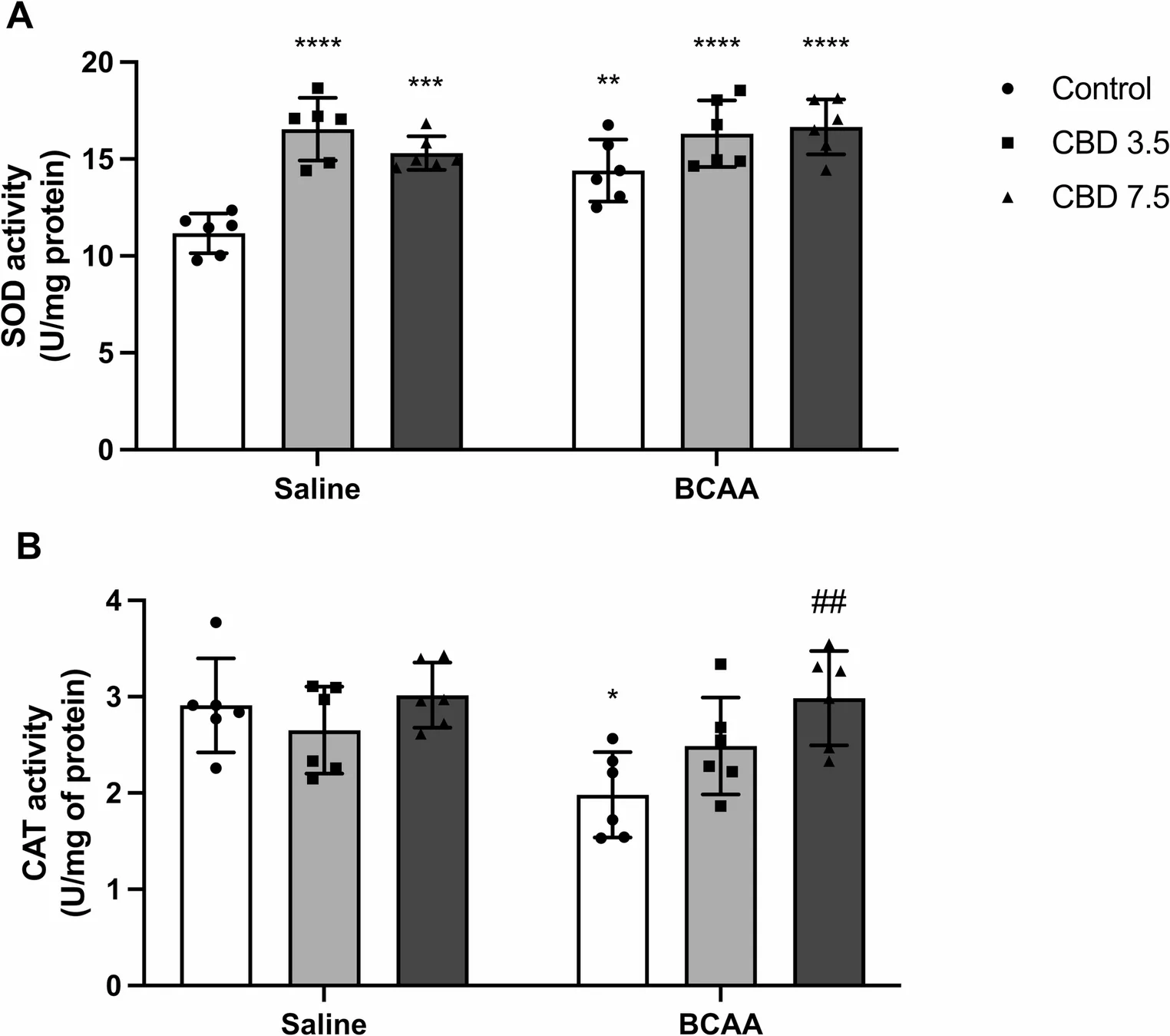
Effects of BCAA administration and CBD treatment on antioxidant enzyme activity in rat cortex. (**A**) Superoxide dismutase (SOD) activity. (**B**) Catalase (CAT) activity. Experimental groups included saline control, saline CBD 3.5 mg/kg, saline CBD 7.5 mg/kg, BCAA control, BCAA + CBD 3.5 mg/kg, and BCAA + CBD 7.5 mg/kg. Data are presented as mean ± standard deviation (SD), with *n* = 6 animals per group. Statistical analysis was performed using ordinary two-way ANOVA followed by Tukey’s multiple-comparisons test, *p* < 0.05, *p < 0.01, **p < 0.001, ***p < 0.0001 vs. saline control group; #*p* < 0.05, ##*p* < 0.01, ###*p* < 0.001, ####*p* < 0.0001 vs. BCAA control group

Furthermore, Fig. 7A shows that SOD activity exhibited significant interaction (*p* = 0.0190), experimental condition (*p* = 0.0044), and treatment (*p* < 0.0001) effects. SOD activity was increased in all experimental groups compared with the control group, including the CBD 3.5 (*p* < 0.0001), CBD 7.5 (*p* = 0.0002), BCAA control (*p* = 0.0050), BCAA + CBD 3.5 (*p* < 0.0001), and BCAA + CBD 7.5 (*p* < 0.0001) groups. Figure 7B shows that CAT activity exhibited significant interaction (*p* = 0.0469), experimental condition (*p* = 0.0199), and treatment (*p* = 0.0145) effects. CAT activity was reduced in the BCAA control group compared with the control group (*p* = 0.0157), whereas treatment with CBD 7.5 restored CAT activity compared with the BCAA control group (*p* = 0.0076).

## Discussion

In this study, we aimed to investigate whether oral administration of a full-spectrum CBD-rich oil could modulate memory performance and cholinergic function in a rat model of MSUD. Our behavioral findings show that neither dose affected locomotion or exploratory activity in open-field test, indicating no nonspecific motor effects. In the object recognition task, the 3.5 mg/kg dose facilitated STM formation in animals submitted to BCAA administration, whereas the 7.5 mg/kg dose preserved STM only in saline-treated animals, without reversing the BCAA-induced impairment. These results indicate that BCAA administration can alter memory in both the short and long term. However, the animals treated with CBD 7.5 did not show differences in STM or LTM, suggesting no memory impairment, unlike the BCAA group. Also, the CBD treatment only reverses the memory damage in STM at 3.5 mg/kg and does not improve the LTM. However, we need to observe that this is the first work using cannabis-related products in MSUD. Moreover, we selected 3.5 and 7.5 mg/kg based on a pilot study; both doses showed greater effects, but lower doses may be preferable, given that we are using neonates. At the neurochemical level, both doses decreased AChE and increased choline acetyltransferase activity in cerebral cortex, accompanied by reduced oxidative damage—as evidenced by lower TBARS and DCFH levels—and an attenuation of inflammatory markers. Collectively, these findings indicate that full-spectrum CBD exerts dose-dependent effects on memory and provides neurochemical protection in neonate rat MSUD model.

Studies have shown that the accumulation of leucine, isoleucine, and valine leads to many problems in the CNS due to increased lactate formation from impaired nicotinamide adenine dinucleotide (NAD) oxidation, resulting in reduced adenosine triphosphate (ATP) formation. Also, the reduction of glutamate concentrations in the brain by branched-chain amino acid transaminase activity depletes glutamate, leading to the formation of alpha-ketoglutarate; collectively, these changes can disturb plasticity, affect memory and learning in animal models of MSUD (Amaral et al. 2010; da Silva Lemos et al. 2022; Strauss et al. 2020). In addition to glutamate depletion in the brain, other neurotransmitters are present at low levels in MSUD, including dopamine, serotonin, and norepinephrine. Attributable to decreased essential amino acids by competition across the blood-brain barrier and related to the appearance of mood and cognition alterations. Thus, it is observed that neurotransmitters, receptors, and synaptic changes are associated with a high risk of developing neuropsychiatric diseases in MSUD patients (Muelly et al. 2013; Zinnanti et al. 2009). Characterizing these alterations in patients is essential to improving our understanding of the pathophysiology and clinical progression of MSUD, particularly the neurological and metabolic impairments associated with BCAA accumulation. However, because MSUD is a rare inherited disorder and studies involving human patients—especially neonates and pediatric populations—are inherently limited by ethical, logistical, and recruitment challenges, animal models remain fundamental tools in this field. In this context, experimental models of MSUD are widely used to recapitulate the neurochemical and behavioral damage caused by BCAA accumulation, enabling investigation of disease mechanisms and preclinical evaluation of novel therapeutic approaches to mitigate metabolic, inflammatory, oxidative, and cognitive dysfunctions associated with the disorder (Amaral and Wajner 2022; Lemos et al. 2024; Scaini et al. 2014a).

Furthermore, as cognitive impairment is one of the major outcomes associated with this disorder, the process that leads to memory involves encoding and consolidation; thus, ACh modulates learning in the hippocampus, and the cortex initiates neurotransmission to temporary memory in the hippocampus. After that, these inputs are transferred again to form LTM in cortex (Haam and Yakel 2017). Thus, changes in this neurotransmitter are associated with cognitive and memory damage (Huang et al. 2022). Regarding behavioral tests, our results show no differences in crossing and rearing counts, indicating that animals did not exhibit impaired locomotion or exploration. However, in recognition test, rats in the control group showed STM and LTM memory; in contrast, rats induced with BCAA showed no difference in STM or LTM, indicating memory impairment. According to previous studies, the MSUD model in rats is associated with deficits in memory acquisition in recognition tasks (Lemos et al. 2023) and in inhibitory avoidance (de Castro Vasques et al. 2004), without changes in locomotion or exploration (Scaini et al. 2014a). Herein, it presents like-depressive behavior in sweet food consumption and forced swimming test (Morais et al. 2022; Scaini et al. 2014a). In concordance, when leucine is administered in zebrafish, animals present anxiety-like behavior (Wessler et al. 2019), memory, and social changes (da Silva Lemos et al. 2022; Duarte et al. 2023), as well as a genetic model of MSUD in zebrafish presents motor dysfunction (Friedrich et al. 2012). The authors associated these behavioral alterations with disruptions in specific brain areas, mainly the hippocampus and cortex, and with changes in neurotransmitter levels, specifically glutamate and ACh (da Silva Lemos et al. 2022; Friedrich et al. 2012; Lemos et al. 2023, 2024). Although the open-field results indicate that locomotor and exploratory activity were not altered by the treatments, it is important to consider that behavioral changes in recognition memory tasks may also be influenced by factors such as anxiety-like behavior, motivational state, or subtle emotional alterations. CBD has been reported to exert anxiolytic and motivational effects in various experimental models (Blessing et al. 2015; Campos et al. 2012), which could influence performance in behavioral paradigms involving the exploration of novel objects. Therefore, although the present results are consistent with an effect on cognitive performance, alternative behavioral influences cannot be entirely ruled out and should be considered when interpreting the findings.

Interestingly, the full-spectrum CBD-rich oil at 3,5 mg/kg + saline preserved both STM and LTM, and when the BCAA group was treated with the same dose, it rescued STM memory. In contrast, the 7,5 mg/kg dose preserved STM only in saline-treated animals and did not reverse the BCAA-induced deficit. It is important to note that some studies often cited in the context of cannabinoid effects in developing brains used synthetic agonists rather than CBD. For example, Schneider et al. employed WIN 55,212-2—a potent cannabinoid receptor-1 (CB1) and cannabinoid receptor-2 (CB2) agonist pharmacologically closer to THC than to CBD—and reported behavioral impairments in pubertal rats. Because WIN produces robust CB1 activation, its effects cannot be extrapolated to our findings. In our study, the intervention consisted of a full-spectrum CBD-rich oil, a phytocomplex whose actions reflect the synergistic interplay among cannabinoids, terpenes, and flavonoids—commonly described as the entourage effect. However, because the present study did not include a comparison with purified CBD, it is not possible to determine whether the behavioral effects observed here are specific to the full-spectrum preparation or attributable to CBD alone. Therefore, the potential contribution of phytochemical interactions should be interpreted cautiously and treated as a plausible hypothesis based on prior literature, rather than as a direct demonstration from the present data. This putative synergism has been repeatedly associated with broader anti-inflammatory, antioxidant, and behavioral benefits than those of isolated CBD, as shown in several preclinical studies. Therefore, rather than contradicting our results, the Schneider study highlights that different classes of cannabinoids exert distinct neurodevelopmental effects, underscoring the importance of evaluating full-spectrum preparations separately from synthetic agonists (Schneider et al. 2008). Consistent with these findings, Huffstetler et al. (2023) demonstrated dose-dependent effects of CBD itself in male mice, with lower doses producing more favorable behavioral outcomes. They observed no STM differences between doses (10 and 20 mg/kg) in the recognition test but found reduced LTM performance at lower doses. Further, they found a decrease in LTM in lower doses in wild-type mice compared with vehicle-treated mice (Huffstetler et al. 2023). These findings reinforce the notion that CBD treatment is dose-dependent and that its actions in developing organisms may follow a narrow therapeutic window, which could help explain why the 3.5 mg/kg dose rescued STM in BCAA-treated neonates, whereas the 7.5 mg/kg dose did not yield the same effect (Kruk-Slomka et al. 2017).

Furthermore, a possible explanation for these effects is the relation between N-methyl-D-aspartate (NMDA) and CB1 receptors. A study demonstrates that CB1 receptors modulate synaptic plasticity, in which the endocannabinoid system (ECS) leads to depolarization and activation of NMDA receptors, increasing calcium influx. Given that MSUD pathology itself involves NMDA-mediated excitotoxicity and impaired glutamatergic balance, ECS-mediated modulation of these pathways may partially counteract BCAA-induced disruptions (Hillard 2015). The CB1 receptor is important for LTM, long-term plasticity, and long-term depression in the hippocampus, and is related to the NMDA receptor (Kano 2014). Thus, ECS can regulate NMDA receptors by decreasing receptor activity, suggesting one mechanism of modulation, reflected in glutamate inhibition. Moreover, these ECS are related to emotional homeostasis, acting in the hypothalamic axis and sympathetic system, affecting anxiety and stress. This emotional regulation may involve astroglial CB1 and glutamate signaling (Katona and Freund 2012; Lutz et al. 2015). Also, astroglial CB1 is important for LTM consolidation, acting via NMDA receptors. Thus, when activated, the CB1 receptor enhances long-term synaptic plasticity, dependent on astrocytic calcium signaling and adenosine receptors, which are implicated in memory formation (Gonçalves-Ribeiro et al. 2024; Goodman and Packard 2015). Moreover, CB2 may be involved in cognitive processes, and studies have shown that this receptor is a target for memory disorders associated with the cholinergic system. However, the mechanisms underlying these receptors remain poorly understood (Kruk-Slomka et al. 2022). In this regard, previous studies in our lab have shown changes in the cholinergic system following neonatal BCAA administration. Scaini and colleagues first demonstrated alterations in AChE activity and expression and observed different effects on AChE expression when BCAA was administered acutely (three administrations in rats at 10 and 28 days of life) or chronically (two administrations per day for 21 days, starting at 7 days old). In the acute protocol, increased AChE activity in the hippocampus, striatum, and cerebral cortex, and increased expression in the hippocampus, were observed. In the chronic protocol, alterations in AChE activity across all structures and in RNA expression in the striatum and cerebral cortex were observed, correlating with behavioral alterations in learning and memory observed in MSUD (Scaini et al. 2012a). Here, we observed that neonatal BCAA administration in rats produced results similar to those reported by Scaini et al., altering AChE activity and causing behavioral deficits, suggesting cognitive injury.

It is possible to believe that the decrease in ACh levels may be involved in the cognitive changes observed, since it is well predicted that AChE hydrolyzes ACh, and it is the key enzyme that terminates the action of ACh at cholinergic synapses and is highly efficient in modulating the levels of extracellular ACh and in regulating cholinergic neurotransmission (Scaini et al. 2012a; Tan et al. 2014; Zimmerman and Soreq 2006). In concordance, previous studies have shown increased AChE and decreased ChAT activity in rats and zebrafish induced by MSUD, suggesting that memory and learning impairment are related to cholinergic system changes (Lemos et al. 2023, 2024; Wessler et al. 2019). In this context, studies relating the role of ACh in the CNS participating in the communication by neurons as an excitatory mediator (Picciotto et al. 2012; Sam and Bordoni 2025; Trang and Khandhar 2025), acting in memory, learning, attention, and motivation, also ACh is associated with discriminatory learning in the neocortex and low levels in the hippocampus related to forgetfulness (Haam and Yakel 2017; Trang and Khandhar 2025). Given that CBD modulated both AChE and ChAT activities in this study, it is possible that part of its cognitive effects occur through the restoration of cholinergic homeostasis, a pathway already known to be disrupted in MSUD.

In this line, Abdel-Salam et al. (2016) show that cannabis resin increases AChE activity at 10 and 20 mg/kg in the serum and brain of rats. However, at 5 mg/kg, it did not differ from the saline group. These results may vary by the administration route and treatment time, as the differences between cannabis extract concentrations can be explained. Once Abdel-Salam presents 20% of THC subcutaneously, we use one extract with 0.2% of THC orally. In addition, the animals’ weights and lines were different. In the present study, Wistar rat neonates weighing 10–15 g at birth were used, whereas Abdel-Salam used Sprague-Dawley rats weighing 130–140 g (Abdel-Salam et al. 2016). In contrast, Puopolo et al. report inhibition of AChE activity in vitro at CBD concentrations of 25, 50, 100, and 150 µM (Puopolo et al. 2022). Taken together, these findings suggest that cannabinoid-related modulation of AChE activity may vary according to the phytochemical composition of the preparation, including the relative abundance of THC, CBD, and other constituents. However, because the present study did not include a direct comparison with purified CBD, it is not possible to determine whether the observed cholinergic effects are attributable specifically to the full-spectrum formulation or to CBD itself. Thus, more studies are necessary to evaluate the effects of CBD on AChE activity, while accounting for oil type, cannabinoid concentration, treatment duration, lineage, and animal age.

Using a neonatal animal model of MSUD, Lemos and colleagues demonstrated that memantine treatment improved oxidative stress, inflammation, the cholinergic system, and memory impairment (Lemos et al. 2023). Importantly, the relevance of this finding lies in the fact that cognitive dysfunction is a parameter altered in the MSUD model, as is observed in other neurological disorders. Memantine, an NMDA receptor antagonist commonly used in Alzheimer’s disease (AD), has been associated with neuroprotective effects, particularly because hyperstimulation of NMDA receptors has been linked to cholinergic neuron death and consequent memory impairment in AD (Parsons et al. 2013). Further, a recent study demonstrated that BCAAs in MSUD can form amyloid-like structures, thereby increasing inflammation and oxidative stress. Since amyloid plaque formation is a hallmark pathological feature of Alzheimer’s disease (AD) and is strongly associated with cognitive impairment, these findings suggest a potential mechanistic link between MSUD-related neurotoxicity and AD-like pathological processes (Kreiser et al. 2023). Thus, cytokines are essential for proliferation, differentiation, and immune cell survival. In this regard, cytokines can be inflammatory or anti-inflammatory; inflammatory cytokines can increase inflammation by activating macrophages, natural killer cells, and lymphocytes. The anti-inflammatory will attenuate inflammatory responses by reducing pro-inflammatory mediators and suppressing monocyte activity. The results presented here demonstrated higher levels of IL-1β and IL-6, with no alteration in TNF-α. According to these results, the increase in pro-inflammatory cytokines in BCAA group reinforces the finding that inflammation is a mechanism underlying the disease-related damage. Corroborating this, Rosa et al. (2016) reported alterations in inflammatory cytokines, including decreased anti-inflammatory cytokines, in an acute model of MSUD (Rosa et al. 2016). Further, Scaini et al. (2014) demonstrated in an animal model of MSUD that inflammation was associated with blood-brain barrier alterations, suggesting that alterations in inflammatory parameters and oxidative stress could increase blood-brain barrier permeability, thereby exacerbating brain dysfunction (Scaini et al. 2014b). Thus, to accelerate enzymatic defense and stimulate immune cells, the organism increases inflammatory responses, which may, in turn, increase reactive species and alter cerebral homeostasis, thereby compromising neuronal function (Scaini et al. 2018).

In this regard, studies have shown that neurological damage and oxidative stress are involved in MSUD, with excess reactive species and reduced antioxidant defenses contributing to tissue damage (Barschak et al. 2008b; Sitta et al. 2014). In the present study, increases in reactive species (measured by DCFH), lipid peroxidation (measured by TBARS levels), and protein damage (measured by sulfhydryl content) were observed. In this line, it was previously established that MSUD-generated metabolites contribute to oxidative damage and disorganized antioxidant defenses. To counter oxidative damage from reactive species, the organism has an antioxidant system comprising enzymes such as SOD and CAT. These enzymes were altered by BCAA administration, with increased SOD activity and decreased CAT activity. Importantly, the oxidative stress profile observed here suggests not only increased production of reactive species but also a dysregulated antioxidant response. The increase in SOD activity accompanied by a reduction in CAT activity may indicate a compensatory response to superoxide overproduction that is not followed by efficient hydrogen peroxide detoxification. Since SOD converts superoxide radicals into hydrogen peroxide, reduced CAT activity may favor hydrogen peroxide accumulation, thereby promoting secondary oxidative damage through lipid peroxidation and protein oxidation (Fukai and Ushio-Fukai 2011). This imbalance between antioxidant enzymes may help explain the differential alterations observed among DCFH, TBARS, and sulfhydryl measurements, suggesting that oxidative damage in the MSUD model involves a complex disruption of redox homeostasis rather than a uniform increase in oxidative markers. The distinct changes among oxidative markers may also reflect differential susceptibility of cellular components to oxidative injury (Muralidharan and Mandrekar 2013). Further, increased TBARS levels suggest enhanced membrane lipid damage, whereas changes in sulfhydryl content indicate oxidation of protein thiol groups, which may directly affect enzyme function and neuronal signaling pathways. Such redox imbalance may contribute to mitochondrial dysfunction, synaptic impairment, and neuronal vulnerability, which are consistent with the behavioral and cognitive alterations observed in the MSUD model (Lemos et al. 2024; Sitta et al. 2014). Moreover, these results were consistent with previous studies reporting oxidative stress and alterations in antioxidant levels after BCAA administration (da Silva Lemos et al. 2025; Lemos et al. 2024).

So, new approaches are essential since MSUD is a rare disease, based on diet restriction, and the discovery of new drugs could improve the neurochemical damage and help to understand the molecular changes in this disease. In this context, studies have shown that cannabinoids can act in many tissues and systems, affecting their pathways. For this reason, they have emerged as a research target for their analgesic effects and their modulation of the immune system, apoptosis, learning, and memory processes. Where some ligands of cannabinoid (CB) receptors can interact with many systems of the CNS, like dopaminergic, glutamatergic, serotonergic, and cholinergic systems (Hillard 2015; Pagano et al. 2022). Thus, CB ligands may act through CB1 and CB2 receptors, which are mainly expressed in neurons and immune system cells, respectively (Gobira et al. 2024; Hillard 2015). Studies have shown that CB1 receptors are related to the psychoactive effects of Cannabis (Alves et al. 2020) and also regulate cell differentiation and proliferation. In the brain, they mainly modulate synaptic plasticity and regulate neurotransmitter release (Bara et al. 2021; Freund et al. 2003). In addition to receptor-mediated actions, recent evidence highlights that full-spectrum CBD-rich preparations may exert broader neurobiological effects through the so-called “entourage effect.” This synergistic interaction among cannabinoids, terpenes, and flavonoids has been shown to enhance anti-inflammatory, antioxidant, and neuroprotective effects beyond those observed with purified CBD alone. Supporting this view, Ribeiro de Novais Junior et al. (2024) demonstrated that a full-spectrum extract more effectively reversed inflammation-induced depressive-like behavior and hypolocomotion than isolated CBD, potentially due to multi-target modulation of cytokine and redox systems. Similarly, Stork et al. (2025) reported that a full-spectrum *Cannabis sativa* extract improved gut–brain–peripheral organ integrity following ischemic injury, reinforcing the hypothesis that phytochemical synergism contributes to functional recovery (de Souza Stork et al. 2025). Considering these findings, the cholinergic, antioxidant, and anti-inflammatory improvements observed in the present MSUD model may, at least in part, reflect these synergistic interactions inherent to full-spectrum formulations.

Furthermore, the ECS is involved in calcium blockade via NMDA receptor antagonism, immune function, stress regulation, and cognitive processes. Also, studies have shown ECS signaling dysregulation may be linked with neurodegenerative and psychiatric processes (Hillard 2015; Katona and Freund 2012; Śmiarowska et al. 2022). Also, the CBD has immunosuppressive and anti-inflammatory effects by suppressing pro-inflammatory cytokines and enhancing anti-inflammatory responses (Jean-Gilles et al. 2015; Santiago et al. 2019). These results are observed in the full-spectrum CBD-rich oil treatment in this study, suggesting that CBD’s anti-inflammatory actions extend to conditions of metabolic neurotoxicity such as MSUD. CBD can decrease elevated levels of IL-1β, IL-6, and TNF-α, potentially reducing BCAA-induced inflammation, which may be related to microglial activation and the restoration of redox balance. The anti-inflammatory properties of CBD may be related to the cortical reduction of TNF-α, which inhibits its expression and reduces inflammatory parameters (Pereira et al. 2021). Also, the CBD could act as a reactive species in generation of TNF-α, either directly or indirectly. The activation of CB1 receptors could be associated with an increase in pro-inflammatory responses through TNF-α synthesis and the generation of reactive species; in contrast, the activation of CB2 receptors leads to an inverse effect, decreasing reactive species and TNF-α, which is essential for reducing inflammation and oxidative stress (Atalay et al. 2019). CBD also reduces reactive species production by altering redox balance, for example, by regulating transcription factors such as Nrf2 in microglia, reducing leukocyte metabolism, or upregulating enzymes such as SOD and components of the glutathione cycle (Jastrząb et al. 2019; Pereira et al. 2021).

This work presents results showing that both doses of a full-spectrum, CBD-rich oil improve the cholinergic system and reduce oxidative stress and inflammation. Still, these effects did not translate into behavioral tests or antioxidant activities. Also, the studies using CBD present discrepancies in the data related to route administration, THC concentration, treatment time, and, mainly, the dose. Worth highlighting that this is the first work that uses CBD treatment for MSUD in a neonate model; more studies are necessary to understand the effects of CBD in neonates, as well as the secure doses and time of treatment. Also, the study used a neonatal Wistar rat model with high-BCAA administration, which should be interpreted as a limitation when extrapolating to the chronic metabolic course of the disease. Also, as an animal model, we cannot observe genetic alterations, developmental progression, and clinical complexity as found in humans. An additional limitation of the present study is the use of only male animals. Sex-dependent differences have been described in neurodevelopmental processes, endocannabinoid signaling, and susceptibility to metabolic and neuroinflammatory insults. Therefore, the present findings should be interpreted within the context of this experimental design. Future studies including both sexes will be important to determine whether the neurochemical and behavioral effects observed here are sex dependent.

## Conclusion

In summary, full-spectrum CBD-rich oil produced significant neurochemical benefits in neonatal MSUD model, including modulation of cholinergic enzymes, reduction of oxidative stress, and attenuation of inflammatory cytokines. Furthermore, treatment was associated with partial and dose-dependent improvements in recognition memory, indicating task-specific effects on STM and LTM performance rather than full memory recovery. These results suggest that the observed protective actions may involve multi-target mechanisms described for phytochemical cannabinoid preparations, although no direct comparison with isolated CBD was conducted. The dose-dependent behavioral profile observed here underscores the importance of identifying therapeutic windows in the development of neural systems. Future studies should explore chronic treatment, long-term neurodevelopmental implications, and mechanistic pathways to further characterize the potential of cannabinoid-based interventions in MSUD and related metabolic encephalopathies.

## Supplementary Information

Below is the link to the electronic supplementary material.

Supplementary Material 1 (PDF 273 KB)

Supplementary Material 2 (PDF 121 KB)

## Data Availability

All data generated or analyzed during this study are included in this published article and available upon request.
