# Supplementary material for: Full-spectrum cannabidiol-rich oil modulates behavior and neurochemical alterations in a rodent model of maple syrup urine disease
Source: Metab Brain Dis. 2026 Sep 3;41(1):204. doi: 10.1007/s11011-026-01958-x (PMC13541999; doi:10.1007/s11011-026-01958-x)
Supplement: Supplementary file 1 [file 11011_2026_1958_MOESM1_ESM.pdf]

Kaycha Labs

4131 SW 47th AVENUE SUITE 1408
DAVIE, FL, 33314, US
(954) 368-7664

Kaycha Labs
N1
Matrix: Edible
Type: Other Edible Product

Certificate of Analysis

COMPLIANCE FOR RETAIL

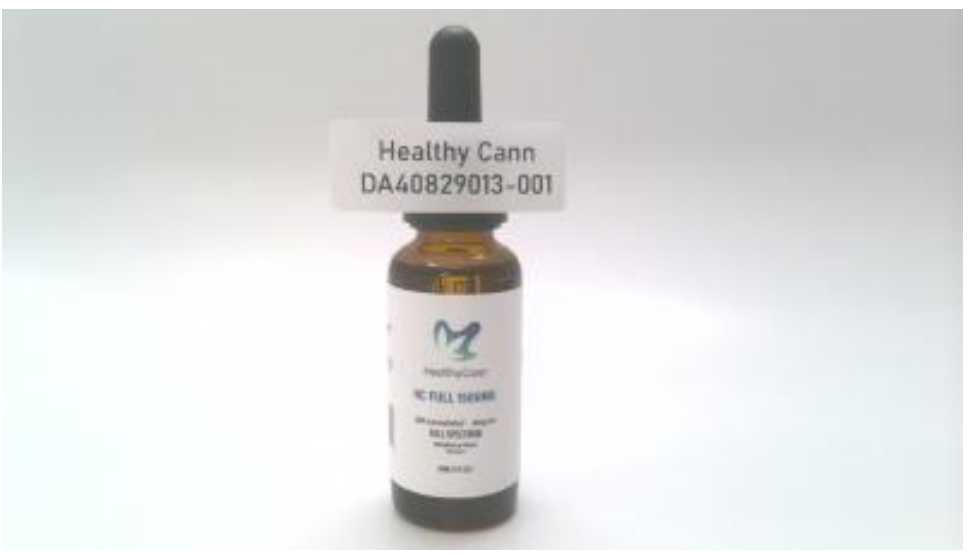

Sample:DA40829013-001
Batch#: T0824-01
Seed to Sale# NA
Batch Date: 08/19/24
Sample Size Received: 28 gram
Total Amount: 1 units
Retail Product Size: 28 gram
Retail Serving Size: 1 gram
Servings: 28
Ordered: 08/28/24
Sampled: 08/29/24
Completed: 09/02/24
Sampling Method: SOP.T.20.010.FL

Sep 02, 2024 | Healthy Cann
3925 N I-10 Service Rd W Ste 223
Metairie, LA, 70002, US

PASSED

SAFETY RESULTS

Pesticides	Heavy Metals	Microbials	Mycotoxins	Residuals Solvents	Filth	Water Activity	Moisture
PASSED	PASSED	PASSED	PASSED	PASSED	PASSED	NOT TESTED	NOT TESTED

MISC.

Terpenes
TESTED

Cannabinoid

PASSED

Total THC
0.177%
Total THC/Container : 49.560 mg

Total CBD
5.451%
Total CBD/Container : 1526.280 mg

Total Cannabinoids
6.032%
Total Cannabinoids/Container : 1688.960 mg

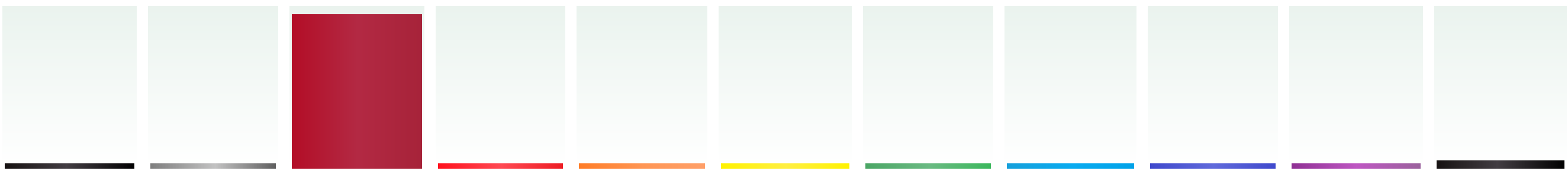

	D9-THC	THCA	CBD	CBDA	D8-THC	CBG	CBGA	CBN	THCV	CBDV	CBC
%	0.177	ND	5.451	ND	ND	0.072	ND	0.024	ND	0.048	0.260
mg/unit	49.56	ND	1526.28	ND	ND	20.16	ND	6.72	ND	13.44	72.80
LOD	0.001 %	0.001 %	0.001 %	0.001 %	0.001 %	0.001 %	0.001 %	0.001 %	0.001 %	0.001 %	0.001 %

Analyzed by: 3335, 1665, 585, 1440
Weight: 3.0967g
Extraction date: 08/29/24 14:23:07
Extracted by: 3335

Analysis Method : SOP.T.40.031, SOP.T.30.031
Analytical Batch : DA077432POT
Instrument Used : DA-LC-007
Analyzed Date : 08/29/24 14:39:46

Reviewed On : 08/30/24 13:12:00
Batch Date : 08/29/24 11:09:16

Dilution : 40
Reagent : 061724.01; 082724.R04; 071124.23; 071624.04; 081524.R03
Consumables : 947.109; 021824CH01; CE0123; R1KB14270
Pipette : DA-079; DA-108; DA-078

Full Spectrum cannabinoid analysis utilizing High Performance Liquid Chromatography with UV detection in accordance with F.S. Rule 64ER20-39.

This Kaycha Labs Certification shall not be reproduced, unless in its entirety, without written approval from Kaycha Labs. The results relate only to the material or product analyzed. ND=Not Detected, ppm=Parts Per Million, ppb=Parts Per Billion, RSD=Relative Standard Deviation. Limit of Detection (LOD) and Limit Of Quantitation (LOQ) are terms used to describe the smallest concentration that can be detected and reliably measured by an analytical procedure, respectively. Action Levels are State determined thresholds based on F.S. Rule 64ER20-39 and F.S. Rule 5K-4. The Measurement of Uncertainty (MU) error is available from the lab upon request. The "Decision Rule" for pass/fail does not include the MU. Any calculated totals may contain rounding errors.

Vivian Celestino
Lab Director

State License # CMTL-0002
ISO 17025 Accreditation # ISO/IEC 17025:2017 Accreditation PJLA-Testing 97164

Signature
09/02/24

4131 SW 47th AVENUE SUITE 1408
DAVIE, FL, 33314, US
(954) 368-7664

Kaycha Labs

N1
Matrix : Edible
Type: Other Edible Product

Certificate of Analysis

PASSED

Healthy Cann

3925 N I-10 Service Rd W Ste 223
Metairie, LA, 70002, US
Telephone: (337) 344-1797
Email: usaoperations@healthycannbr.com

Sample : DA40829013-001
Batch# : T0824-01
Sampled : 08/29/24
Ordered : 08/29/24

Sample Size Received : 28 gram
Total Amount : 1 units
Completed : 09/02/24 **Expires:** 09/02/25
Sample Method : SOP Client Method

Terpenes

TESTED

Terpenes	LOD (%)	mg/unit	%	Result (%)
TOTAL TERPENES	0.007	7.56	0.027	
ALPHA-BISABOLOL	0.007	7.56	0.027	
3-CARENE	0.007	ND	ND	
BORNEOL	0.013	ND	ND	
CAMPHENE	0.007	ND	ND	
CAMPHOR	0.007	ND	ND	
CARYOPHYLLENE OXIDE	0.007	ND	ND	
CEDROL	0.007	ND	ND	
EUCALYPTOL	0.007	ND	ND	
FARNESENE	0.007	ND	ND	
FENCHONE	0.007	ND	ND	
FENCHYL ALCOHOL	0.007	ND	ND	
GERANIOL	0.007	ND	ND	
GERANYL ACETATE	0.007	ND	ND	
GUAIOL	0.007	ND	ND	
HEXAHYDROTHYMOL	0.007	ND	ND	
ISOBORNEOL	0.007	ND	ND	
ISOPULEGOL	0.007	ND	ND	
LIMONENE	0.007	ND	ND	
LINALOOL	0.007	ND	ND	
NEROL	0.007	ND	ND	
OCIMENE	0.007	ND	ND	
PULEGONE	0.007	ND	ND	
SABINENE	0.007	ND	ND	
SABINENE HYDRATE	0.007	ND	ND	
VALENCENE	0.007	ND	ND	
ALPHA-CEDRENE	0.005	ND	ND	
ALPHA-HUMULENE	0.007	ND	ND	
ALPHA-PHELLANDRENE	0.007	ND	ND	
ALPHA-PINENE	0.007	ND	ND	
ALPHA-TERPINENE	0.007	ND	ND	
ALPHA-TERPINEOL	0.007	ND	ND	
ALPHA-TERPINOLENE	0.007	ND	ND	
BETA-CARYOPHYLLENE	0.007	ND	ND	
BETA-MYRCENE	0.007	ND	ND	
BETA-PINENE	0.007	ND	ND	
CIS-NEROLIDOL	0.003	ND	ND	
GAMMA-TERPINENE	0.007	ND	ND	
TRANS-NEROLIDOL	0.005	ND	ND	

Analyzed by: 4451, 3605, 585, 1440
Weight: 0.1994g
Extraction date: 08/29/24 13:28:41
Extracted by: 4451

Analysis Method : SOP.T.30.061A.FL, SOP.T.40.061A.FL
Analytical Batch : DA077411TER
Instrument Used : DA-GCMS-008
Analyzed Date : 08/29/24 13:28:49
Reviewed On : 09/01/24 10:42:38
Batch Date : 08/29/24 10:04:48

Dilution : 10
Reagent : 022224.04
Consumables : 947.109; 240321-634-A; 280670723; CE0123
Pipette : DA-065

Terpenoid testing is performed utilizing Gas Chromatography Mass Spectrometry. For all Flower samples, the Total Terpenes % is dry-weight corrected.

Total (%) 0.027

This Kaycha Labs Certification shall not be reproduced, unless in its entirety, without written approval from Kaycha Labs. The results relate only to the material or product analyzed. ND=Not Detected, ppm=Parts Per Million, ppb=Parts Per Billion, RSD=Relative Standard Deviation. Limit of Detection (LOD) and Limit Of Quantitation (LOQ) are terms used to describe the smallest concentration that can be detected and reliably measured by an analytical procedure, respectively. Action Levels are State determined thresholds based on F.S. Rule 64ER20-39 and F.S. Rule 5K-4. The Measurement of Uncertainty (MU) error is available from the lab upon request. The "Decision Rule" for pass/fail does not include the MU. Any calculated totals may contain rounding errors.

Vivian Celestino
Lab Director

State License # CMTL-0002
ISO 17025 Accreditation # ISO/IEC 17025:2017 Accreditation PJLA-Testing 97164

Signature
09/02/24
