# Supplementary material for: Full-spectrum cannabidiol-rich oil modulates behavior and neurochemical alterations in a rodent model of maple syrup urine disease
Source: Metab Brain Dis. 2026 Sep 3;41(1):204. doi: 10.1007/s11011-026-01958-x (PMC13541999; doi:10.1007/s11011-026-01958-x)
Supplement: Supplementary file 2 [file 11011_2026_1958_MOESM2_ESM.pdf]

Table S1. Detailed statistical analyses for behavioral, cholinergic, inflammatory, and oxidative stress parameters.

Parameter	Statistical analysis	Interaction effect	Experimental effect	Treatment effect	Significant post hoc comparisons / main findings
**Open-field: Crossings**	One-way ANOVA	—	—	F(5,96)=1.257, p=0.290	No significant differences among groups
**Open-field: Rearings**	One-way ANOVA	—	—	F(5,96)=1.455, p=0.212	No significant differences among groups
**NOR training session**	One-way ANOVA	—	—	p=0.172	No differences among groups during training
**NOR STM**	Paired t-test	—	—	—	Increased recognition index in Control (t=-5.039, df=10, p=0.001), CBD 3.5 (t=-3.482, df=18, p=0.003), CBD 7.5 (t=-2.221, df=17, p=0.040), and BCAA+CBD 3.5 (t=-2.875, df=17, p=0.011). No difference in BCAA (t=-0.582, df=14, p=0.570) or BCAA+CBD 7.5 (t=-1.374, df=15, p=0.190).
**NOR LTM**	Paired t-test	—	—	—	Increased recognition index in Control (t=-3.616, df=10, p=0.005) and CBD 3.5 (t=-2.309, df=19, p=0.033). No difference in BCAA (t=1.274, df=14, p=0.223) or BCAA+CBD 7.5 (t=-1.142, df=15, p=0.271).
**ChAT activity**	Two-way ANOVA	F(2,30)=15.62, p<0.0001	F(1,30)=2.022, p=0.1653	F(2,30)=5.816, p=0.0073	BCAA decreased activity vs Control (MD=1.756; 95% CI=0.069–3.444; p=0.0479). CBD 3.5 and CBD 7.5 increased activity vs BCAA (MD=-1.912, CI=-3.599 to -0.225, p=0.0192; MD=-3.523, CI=-5.210 to -1.835, p<0.0001). BCAA+CBD 7.5 increased activity vs Control (MD=-1.766, CI=-3.454 to -0.079, p=0.0360).
**AChE activity**	Two-way ANOVA	F(2,30)=5.087, p=0.0125	F(1,30)=18.84, p=0.0001	F(2,30)=28.43, p<0.0001	BCAA increased AChE vs Control (MD=-4.690, CI=-8.255 to -1.124, p=0.0047). CBD 3.5 and CBD 7.5 restored activity vs BCAA (MD=8.081, CI=4.516–11.65, p<0.0001; MD=5.324, CI=1.759–8.890, p=0.0024).
**IL-1β**	Two-way ANOVA	F(2,30)=15.52, p<0.0001	F(1,30)=3.063, p=0.0903	F(2,30)=38.21, p<0.0001	BCAA increased IL-1β vs Control (MD=-32.78, CI=-61.47 to -4.085, p=0.0179). CBD treatments reduced cytokine levels vs BCAA (CBD 3.5: MD=86.35, CI=57.65–115.0, p<0.0001; CBD 7.5: MD=70.94, CI=42.24–99.63, p<0.0001).
**IL-6**	Two-way ANOVA	F(2,30)=8.955, p=0.0009	F(1,30)=0.6668, p=0.4206	F(2,30)=9.587, p=0.0006	BCAA increased IL-6 vs Control (MD=-1.106, CI=-2.039 to -0.1733, p=0.0129). CBD 3.5 reversed this effect (MD=1.748, CI=0.8152–2.681, p<0.0001).
**TNF-α**	Two-way ANOVA	F(2,30)=0.7427, p=0.4844	F(1,30)=2.400, p=0.1318	F(2,30)=12.65, p=0.0001	CBD 3.5 and CBD 7.5 reduced TNF-α vs Control (MD=0.08483, CI=0.01966–0.1500, p=0.0052; MD=0.07500, CI=0.009824–0.1402, p=0.0168) and vs BCAA group.
**DCFH oxidation**	Two-way ANOVA	F(2,30)=3.205, p=0.0548	F(1,30)=3.065, p=0.0902	F(2,30)=14.19, p<0.0001	BCAA increased oxidative damage vs Control (MD=-0.1577, CI=-0.3139 to -0.00152, p=0.0467). CBD 3.5 and CBD 7.5 reversed this effect.
**TBARS**	Two-way ANOVA	F(2,30)=7.287, p=0.0026	F(1,30)=0.0005, p=0.9815	F(2,30)=10.96, p=0.0003	BCAA increased TBARS vs Control (MD=-0.1782, CI=-0.3519 to -0.00445, p=0.0418). CBD 3.5 and CBD 7.5 reduced TBARS levels.
**Sulfhydryl content**	Two-way ANOVA	F(2,30)=1.564, p=0.2258	F(1,30)=11.41, p=0.0020	F(2,30)=7.403, p=0.0024	Reduced levels in CBD 7.5, BCAA, BCAA+CBD 3.5, and BCAA+CBD 7.5 compared with Control.
**SOD activity**	Two-way ANOVA	F(2,30)=4.538, p=0.0190	F(1,30)=9.508, p=0.0044	F(2,30)=23.67, p<0.0001	Increased SOD activity in all experimental groups compared with Control (p≤0.0050).
**CAT activity**	Two-way ANOVA	F(2,30)=3.393, p=0.0469	F(1,30)=6.051, p=0.0199	F(2,30)=4.895, p=0.0145	BCAA decreased CAT activity vs Control (MD=0.9277, CI=0.1277–1.728, p=0.0157). CBD 7.5 restored CAT activity vs BCAA (MD=-1.004, CI=-1.804 to -0.2037, p=0.0076).

Abbreviations: ANOVA, analysis of variance; MD, mean difference; CI, confidence interval; NOR, novel object recognition; STM, short-term memory; LTM, long-term memory; ChAT, choline acetyltransferase; AChE, acetylcholinesterase; DCFH, 2′,7′-dichlorofluorescein oxidation; TBARS, thiobarbituric acid reactive substances; SOD, superoxide dismutase; CAT, catalase.
